# Long-Term Effects of Opium Consumption Following Percutaneous Coronary Intervention: A 10-year Follow-Up Study

**DOI:** 10.5334/gh.1315

**Published:** 2024-04-24

**Authors:** Ali Izadi Amoli, Alireza Oraii, Faezeh Aghajani, Mana Jameie, Zahra Lotfi, Arash Jalali, Akbar Shafiee, Mohammad Sadeq Najafi, Masoumeh Lotfi-Tokaldany, Seyedeh Hamideh Mortazavi, Mojgan Ghavami, Ignacio J. Amat-Santos, Mohammad Hadi Mansouri, Hassan Aghajani

**Affiliations:** 1 Tehran Heart Center, Cardiovascular Diseases Research Institute, Tehran University of Medical Sciences, Tehran, Iran; 2 Interventional Cardiology Research Center, Cardiovascular Research Institute, Isfahan University of Medical Sciences, Isfahan, Iran; 3 Cardiac Primary Prevention Research Center, Cardiovascular Diseases Research Institute, Tehran University of Medical Sciences, Tehran, Iran; 4 CIBERCV, Cardiology Department, University Hospital of Valladolid, Valladolid, Spain

**Keywords:** Percutaneous coronary intervention, coronary artery disease, opium, major adverse cardiac and cerebrovascular event, all-cause mortality

## Abstract

**Background:**

Opium consumption has been an overlooked health issue in the Iranian population, and the prognostic role of opium consumption in patients undergoing coronary revascularization is unknown.

**Hypothesis:**

We aimed to assess the association between opium consumption and long-term cardiovascular outcomes after percutaneous coronary intervention (PCI).

**Methods:**

We screened 2203 consecutive patients who underwent elective PCI between April 2009 and April 2010 at Tehran Heart Center. Exclusion criteria were unsuccessful PCI, non-elective PCI, and missing opium use data. Opium consumption was defined as self-reported ever use of any traditional opium substances. Outcomes of interest were all-cause mortality and a composite of major adverse cardiac and cerebrovascular events (MACCE). The association between opium use and study outcomes was evaluated using the inverse probability of treatment weighting (IPTW) method. Cumulative hazard curves were demonstrated to further assess the association visually. Furthermore, the effect of opium consumption on individual components of MACCE was evaluated in a competing risk setting.

**Results:**

A total of 2025 elective PCI patients were included (age: 58.7 ± 10.67, 29.1% women), among whom 297 (14.6%) patients were opium users. After a median follow-up of 10.7 years, opium consumption was associated with a higher risk of all-cause mortality (IPTW-hazard ratio [HR] = 1.705, 95% CI: 1.125–2.585; *P* = 0.012) and MACCE (IPTW-HR = 1.578, 95% CI: 1.156–2.153; *P* = 0.004). The assessment of MACCE components suggested a non-significant borderline trend for higher non-fatal myocardial infarction (IPTW–sub-distribution HR [SHR] = 1.731, 95% CI: 0.928–3.231; *P* = 0.084) and mortality (IPTW-SHR = 1.441, 95% CI: 0.884–2.351; *P* = 0.143) among opium users.

**Conclusions:**

Opium consumption is associated with a more than 50% increase in long-term risk of mortality and MACCE in patients undergoing PCI. These findings accentuate the importance of preventive strategies to quit opium addiction in this population.

## Graphical Abstract

**Figure:**
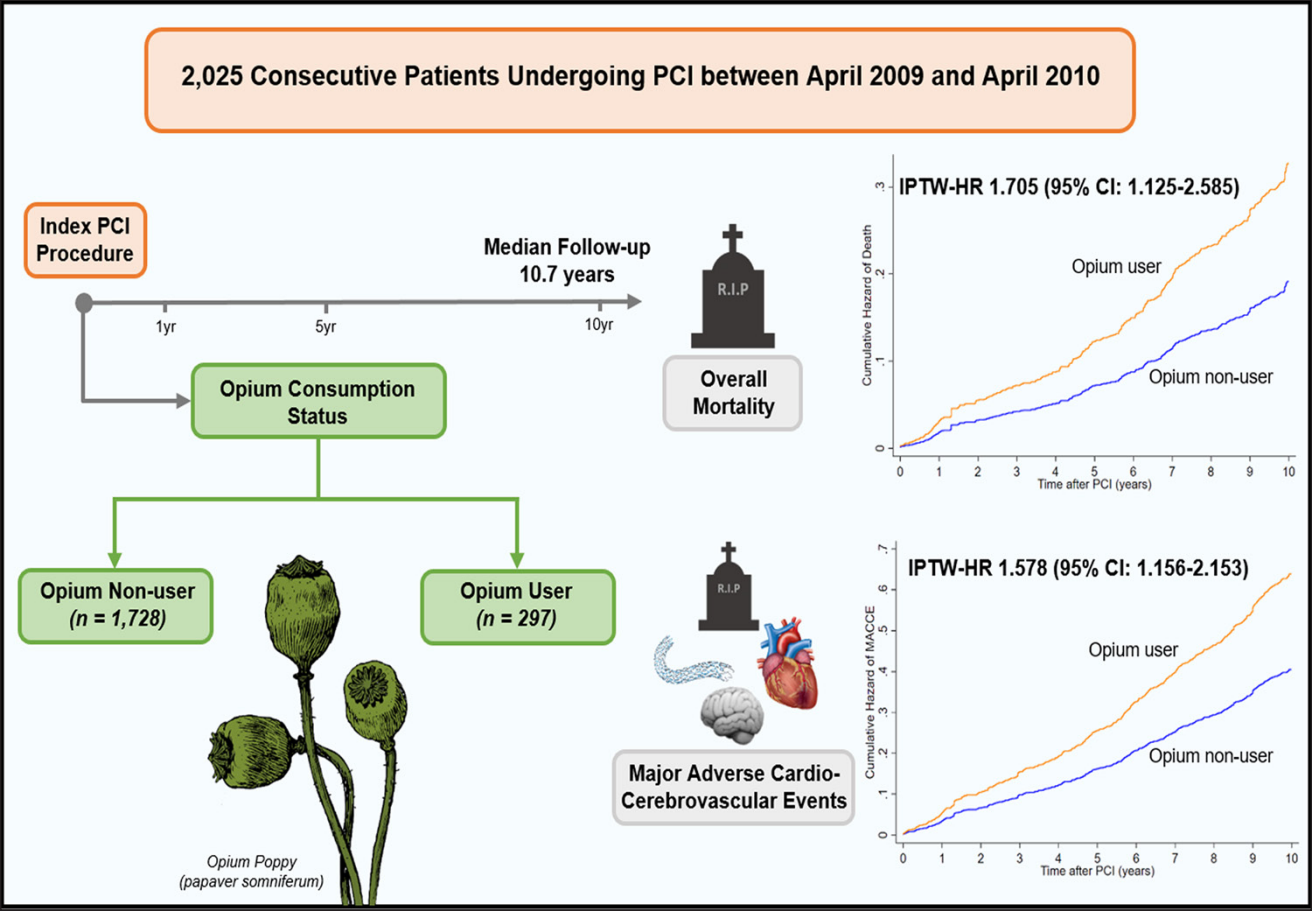
Abbreviations: CI, confidence interval; IPTW, inverse probability of treatment weighting; HR, hazard ratio; PCI, percutaneous coronary intervention

## Introduction

Percutaneous coronary intervention (PCI) is a promising treatment strategy for a wide variety of patients with obstructive coronary artery disease (CAD). A non-negligible number of patients who undergo PCI still experience adverse cardiovascular events in long-term follow-up periods after successful revascularization. Although some studies have explored the predictors of adverse cardiovascular events after PCI, there is still a lack of data on the prognostic role of habitual substance use, specifically opiates, in determining long-term cardiovascular outcomes [1,2,3].

Opium consumption is the second most common drug use disorder worldwide and is even more prevalent among inhabitants of specific geographical regions, such as Middle Eastern countries [4,5]. There are some traditional beliefs that opium has beneficial implications on cardiovascular disease risk factors, such as improving the lipid profile, controlling diabetes mellitus, and even increasing longevity [6]. During the past decade, several studies have tested these traditional hypotheses in the general population and provided equivocal evidence regarding the effect of opium consumption on health outcomes, ischemic cardiovascular events, and mortality [7,8]. While the debate over the potentially harmful effects of opium on the cardiovascular system continues [9], those addicted to opium have a higher risk of all-cause death compared with the general population [10]. Moreover, opium has been identified as an independent risk factor for more complex CAD, as demonstrated in angiographic findings [11,12]. Conversely, in other studies, opium usage was not found to be associated with short-term post-PCI angiographic results and major adverse events [13,14]. Consequently, it is yet unclear whether opium consumption status is an independent factor in determining the long-term prognosis of patients with ischemic heart diseases who require coronary revascularization.

Hence, this study aims to investigate the association between opium consumption and the long-term risk of all-cause mortality and major adverse cardiac and cerebrovascular events (MACCE) in a large cohort of post-PCI patients in a tertiary cardiac center.

## Methods

### Study design and population

This is a registry-based retrospective cohort study using the Coronary Angioplasty Databank of the Tehran Heart Center, a high-volume tertiary cardiac center [15]. Our institutional Review Board and the Ethics Committee of Tehran University of Medical Sciences (IR.TUMS.MEDICINE.REC.1399.051) approved the study design.

Data of all consecutive patients aged ≥18 years who underwent PCI between April 2009 and April 2010 at our center were screened for inclusion. Patients with unsuccessful PCI, non-elective PCI, intravenous thrombolytic therapy before the index procedure, need for urgent coronary artery bypass graft surgery for revascularization, or lack of information on opium consumption status were excluded. The Coronary Angioplasty Databank of the Tehran Heart Center was used to extract data on demographic features, cardiovascular risk factors, previous medical history, lifestyle behaviors, coronary angiographic features, and procedural characteristics. All patients were interviewed regarding their opium consumption habits during the last month before the index procedure. Opium consumption was defined as the self-reported ever use of traditional recreational opium substances either through inhalational (i.e., smoking opium) or oral consumption routes (i.e., chewing opium or drinking opium solutions). Because of unstandardized illegal opium products and an unspecified amount of opium used each time, the data regarding the dosage of opium could not be collected properly.

### Outcomes and follow-up

The primary outcomes were all-cause mortality and a composite of MACCE, defined as a composite of all-cause death, non-fatal myocardial infarction, target lesion and target vessel revascularization, or cerebrovascular events. The individual components of MACCE were further investigated as secondary outcomes. According to our center’s Coronary Angioplasty Follow-up Clinic protocol, all patients were followed through outpatient clinic visits and telephone interviews 1, 3, 6, and 12 months after the index procedure and annually thereafter. More frequent follow-up visits were scheduled for at-risk patients at the discretion of treating physicians. During these follow-up visits, the occurrence of study outcomes and patients’ vital statuses were assessed. In case of an inability to attend in-person visits, the required data were gathered from patients or their designated relatives through telephone interviews. All patients were instructed to provide documented evidence of the adverse events during the in-person visits or send a copy of the requested records for documentation. Adverse events were then confirmed through the review of these documents, complementary imaging, and laboratory investigations.

### Statistical analysis

Categorical variables are reported as numbers (percentages) and were compared between the two groups using the χ^2^ or Fisher exact tests. Continuous variables are presented as mean ± standard deviation (SD) or median (interquartile range [IQR]) and were compared using the *t* test or Mann-Whitney *U* test according to the normality of distribution. The inverse Kaplan-Meier method was used to calculate the median follow-up time. The incidence rates of the primary and secondary outcomes were calculated among opium users and non-users using the Kaplan-Meier method. We further used the stabilized inverse probability of treatment weighting (IPTW) method to assess the effect of opium exposure on study outcomes. This advanced statistical method accounts for the between-group differences observed among opium users and non-users, primarily when an imbalance exists in the risk profile of the two study groups [16]. While stabilized IPTW offers advantages, such as reducing selection bias and enabling direct estimation of causal effects, it also has limitations related to variance estimation, model sensitivity, and implementation complexity [17]. The propensity scores (i.e., probability of exposure to opium use) were created using a logistic regression model including all of the baseline covariates in Table 1, and the IPTWs were then calculated based on propensity scores. The propensity score coverage before and after IPTW modeling is depicted in Supplementary Figure S1. After that, we used a Cox proportional hazard regression model, weighted for the IPTWs, to assess the IPTW-adjusted effect of opium use on all-cause mortality and MACCE in patients undergoing elective PCI. The association between opium use and the individual components of MACCE was assessed similarly in a competing risk setting. In addition, we did a sensitivity analysis to confirm the consistency of our findings using a more conventional method of adjustment in observational studies. We performed a multivariable Cox proportional hazard regression model adjusting for all of the baseline covariates included in Table 1 to assess the adjusted effect of opium use on all-cause mortality and MACCE. Hazard ratios (HRs) and sub-distribution hazard ratios (SHRs) wit h a95% confidence interval (CI) were used to report the effect of opium use on primary and secondary outcomes, respectively. All statistical analyses were performed using IBM SPSS Statistics for Windows, version 25.0 (IBM Corp., Armonk, NY, USA) and STATA (version 14.2).

**Table 1:** Baseline characteristics of the study population.


	OPIUM NON-USERS (n = 1,728)	OPIUM USERS (n = 297)	*p-VALUE*

**Age**, year, mean ± SD	59.3 ± 10.76	54.7 ± 9.21	<0.001

**Female**	579 (33.5%)	11 (3.7%)	<0.001

**BMI**, kg/m^2^	27.8 ± 4.30	26.4 ± 4.00	<0.001

**Diabetes mellitus**	524 (30.3%)	56 (18.9%)	<0.001

**Hypertension**	910 (52.7%)	111 (37.4%)	<0.001

**Dyslipidemia**	1094 (63.3%)	169 (56.9%)	0.035

**Family history of CAD**	327 (19.0%)	53 (18.0%)	0.690

**Cigarette smoker**	359 (20.8%)	178 (59.9%)	<0.001

**eGFR < 60 ml/min**	382 (22.7%)	39 (13.4%)	<0.001

**Left ventricular dysfunction**	167 (10.4%)	36 (12.9%)	0.205

**Prior MI**	847 (49.0%)	174 (58.6%)	0.002

**Prior PCI**	120 (6.9%)	18 (6.1%)	0.577

**Prior CABG**	90 (5.2%)	15 (5.1%)	0.910

**Stable angina**	554 (32.1%)	80 (26.9%)	0.079

**Number of involved vessels**			0.705

SVD	677 (39.2%)	111 (37.4%)	

2VD	679 (39.3%)	116 (39.1%)	

3VD	372 (21.5%)	70 (23.6%)	

**LAD territory involvement**	1111 (64.3%)	167 (56.2%)	0.008

**Drug-eluting stent**	1150 (66.6%)	185 (62.3%)	0.152

**≥2 stents implantation**	461 (26.7%)	69 (23.2%)	0.212


Abbreviations: BMI, body mass index; CABG, coronary artery bypass graft; CAD, coronary artery disease; eGFR, estimated glomerular filtration rate; LAD, left anterior descending coronary artery; MI, myocardial infarction; PCI, percutaneous coronary intervention; SD, standard deviation; SVD, single vessel disease. Data is presented as mean ± SD for continuous and numbers (percentage) for categorical variables.

## Results

**Figure 1:**
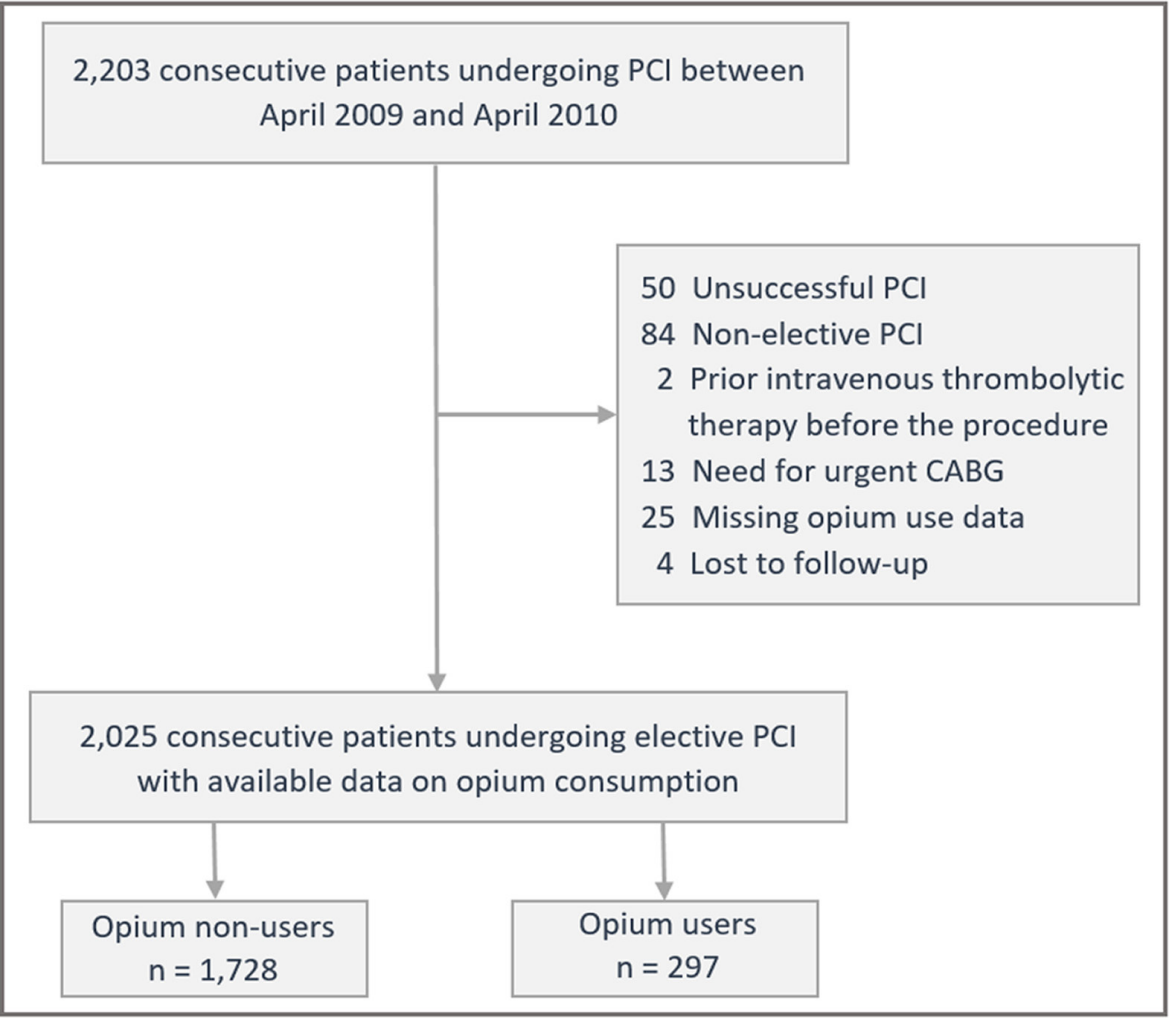
Flow diagram of the study population. Abbreviations: CABG, coronary artery bypass graft; PCI, percutaneous coronary intervention.

A total of 2203 consecutive adult patients underwent PCI between April 2009 and April 2010 at our center. We excluded 178 patients who did not meet the selection criteria and included 2025 patients in this analysis (Figure 1). The mean age of the included patients was 58.7 ± 10.67 years, and 590 (29.1%) patients were women. The baseline characteristics of the study population are shown in Table 1.

Overall, 297 (14.7%) patients reported opium use in our study. Opium users were significantly more likely to be younger and male. Moreover, they had a lower prevalence of diabetes mellitus, hypertension, dyslipidemia, and estimated glomerular filtration rate (<60 mL/min/1.73 m^2^) compared to non-users (Table 1). However, opium users were more likely to be current cigarette smokers and have a prior history of myocardial infarction. There was no significant difference between opium users and non-users regarding angiographic and procedural characteristics, except for a lower rate of left anterior descending coronary territory involvement among opium users (Table 1).

Patients were followed for a median duration of 10.7 years (95% CI: 10.72–10.77), during which 65 (21.9%) opium users and 322 (18.6%) non-users deceased (10-year incidence rate: 22.5 vs. 18.7 per 1000 person-years). Furthermore, 118 (39.7%) opium users and 600 (34.7%) non-users developed MACCE (10-year incidence rate: 46.4 vs. 39.1 per 1000 person-years) during the follow-up period. The analysis of the individual components of MACCE showed that opium users had a higher 10-year incidence of death as the first event (19.5 vs. 18.2 per 1000 person-years), non-fatal myocardial infarction (13.9 vs. 10.2 per 1000 person-years), target vessel/lesion revascularization (10.9 vs. 9.5 per 1000 person-years), and cerebrovascular events (2.2 vs. 1.2 per 1000 person-years) compared to opium non-users (Table 2).

**Table 2:** 10-year incidence rate of cardiovascular outcomes in patients undergoing elective percutaneous coronary intervention, according to opium consumption status.


	OPIUM NON-USERS		OPIUM USERS	
	
	N (%)	10-YEAR INCIDENCE RATE	N (%)	10-YEAR INCIDENCE RATE

**Primary outcomes**				

All-cause mortality	322 (18.6%)	18.7	65 (21.9%)	22.5

MACCE	600 (34.7%)	39.1	118 (39.7%)	46.4

**Secondary outcomes (MACCE components)**				

All-cause death (as the first event)	284 (16.4%)	18.2	52 (17.5%)	19.5

Non-fatal myocardial infarction	148 (8.6%)	10.2	34 (11.4%)	13.9

Target lesion/vessel revascularization	145 (8.4%)	9.5	27 (9.1%)	10.9

Cerebrovascular events	23 (1.3%)	1.2	5 (1.7%)	2.2


Abbreviations: MACCE, major adverse cardiac and cerebrovascular events; 10-year incidence rates are reported as number of events per 1000 person-years. MACCE is the composite of all-cause death, non-fatal myocardial infarction, target lesion/vessel revascularization, or cerebrovascular events.

**Figure 2:**
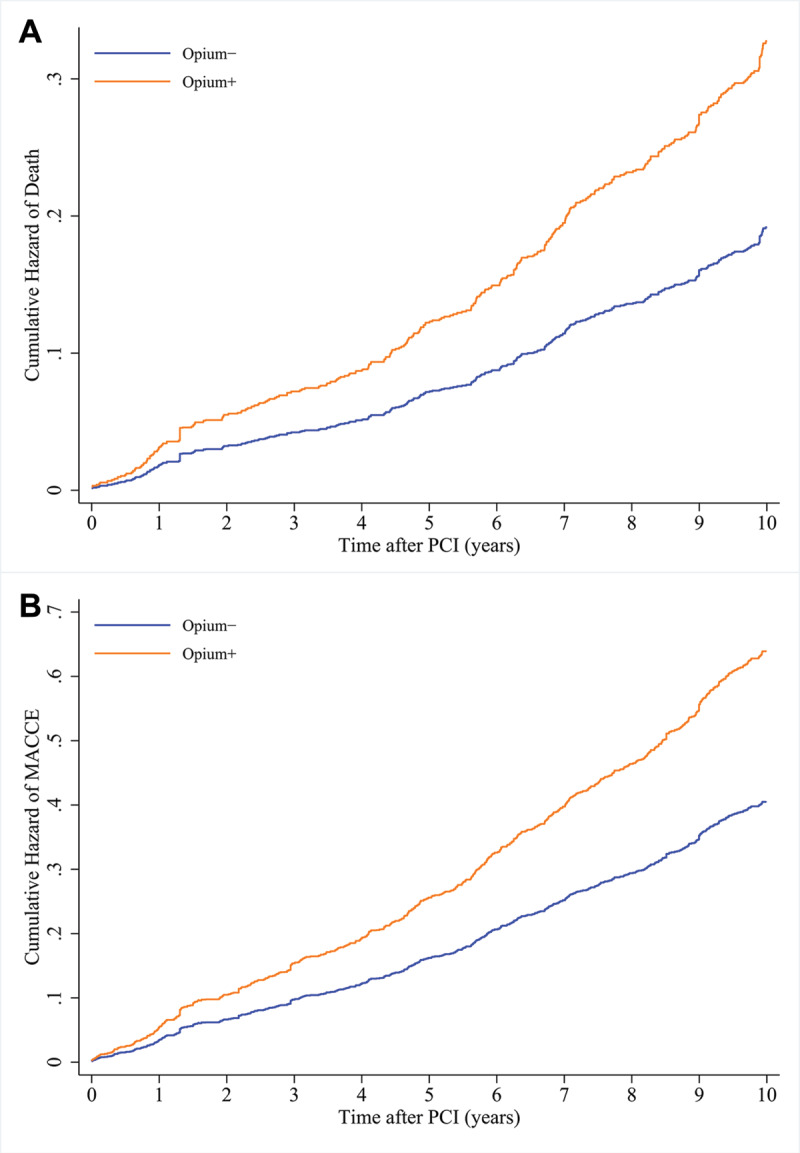
Adjusted cumulative hazard of all-cause mortality **(A)** and major adverse cardio-cerebrovascular events **(B)** in patients with and without opium consumption.

The crude and IPTW-adjusted effects of opium consumption on long-term outcomes of patients undergoing elective PCI are demonstrated in Table 3. The crude effect of opium consumption on the long-term risk of all-cause mortality (crude HR = 1.202, 95% CI: 0.920–1.569; *P* = 0.176) and MACCE (crude HR = 1.189, 95% CI: 0.976–1.449; *P* = 0.085) was non-significant. However, following IPTW adjustment, opium consumption was significantly associated with a higher long-term risk of all-cause mortality (IPTW-HR = 1.705, 95% CI: 1.125–2.585; *P* = 0.012) and MACCE (IPTW-HR = 1.578, 95% CI: 1.156–2.153; *P* = 0.004) during the follow-up period. The adjusted cumulative hazard curves for all-cause mortality and MACCE are shown in Figure 2. In a sensitivity analysis, we further assessed the consistency of these findings using the conventional method of multivariable adjustment. After conventional adjustment for multiple baseline covariates, opium consumption was significantly associated with a higher risk of all-cause mortality (multivariable-adjusted HR = 1.603, 95% CI: 1.185–2.169; *P* = 0.002) and MACCE (multivariable-adjusted HR = 1.277, 95% CI: 1.025–1.519; *P* = 0.029).

**Table 3:** Effect of opium consumption on long-term outcomes of patients undergoing elective percutaneous coronary intervention.


	CRUDE HR (95% CI)	*p-VALUE*	IPTW-HR (95% CI)	*p-VALUE*

**Primary outcomes**				

All-cause mortality	1.202 (0.920–1.569)	0.176	1.705 (1.125–2.585)	**0.012**

MACCE	1.189 (0.976–1.449)	0.085	1.578 (1.156–2.153)	**0.004**

**Secondary outcomes (MACCE components) †**				

All-cause death (as the first event)	1.073 (0.801–1.439)	0.634	1.441 (0.884–2.351)	0.143

Non-fatal myocardial infarction	1.371 (0.944–1.991)	0.097	1.731 (0.928–3.231)	0.084

Target lesion/vessel revascularization	1.123 (0.746–1.690)	0.575	1.114 (0.678–1.828)	0.669

Cerebrovascular events	1.319 (0.501–3.466)	0.574	2.539 (0.523–12.326)	0.247


Abbreviations: CI, confidence interval; HR, hazard ratio; IPTW, inverse probability of treatment weighting; MACCE, major adverse cardiac and cerebrovascular events. MACCE is the composite of all-cause death, non-fatal myocardial infarction, target lesion/vessel revascularization, or cerebrovascular events. † IPTW-SHRs for the individual components of MACCE were calculated in a competing risk setting.

In addition, assessment of the individual components of MACCE in a competing risk setting revealed a non-significant borderline trend for higher non-fatal myocardial infarction (IPTW-SHR = 1.731, 95% CI: 0.928–3.231; *P* = 0.084) and death as the first event (IPTW-SHR. = 1.441, 95% CI: 0.884–2.351; *P* = 0.143) in opium users compared to non-users after IPTW adjustment (Table 3). However, no statistically significant difference in the long-term risk of target vessel/lesion revascularization (IPTW-SHR = 1.114, 95% CI: 0.678–1.828; *P* = 0.669) and cerebrovascular events (IPTW-SHR = 2.539, 95% CI: 0.523–12.326; *P* = 0.247) was observed between opium users and non-users (Table 3).

### Discussion

This study encompassed a large cohort of patients who underwent elective PCI to investigate the effect of traditional opium use on the long-term risk of adverse cardiovascular events during a decade of follow-up. We found that opium consumption was associated with a significantly higher long-term risk of all-cause mortality and MACCE after PCI. However, this association was not observed for MACCE components.

A few studies have investigated the impact of opium consumption on cardiovascular health. One of the earliest pieces of evidence came from a registry-based study that provided valuable insight into the role of opiates as a risk factor for ischemic cardiovascular diseases [7]. Later on, long-term prospective follow-up of a population-based cohort of 50 000 urban/rural dwellers showed that opium users were at a higher risk of death from cardiovascular and cancer-specific causes [18]. This evidence was further complemented in a nested case-control study that showed a higher risk of myocardial infarction among those receiving opiates for pain control [19].

The controversial role of opium addiction on the prognosis of patients with acute myocardial infarctions has been investigated in several studies on the Iranian population, though they have indicated no significant association between opium consumption and short-/mid-term mortality and major adverse events [14,20,21]. Some studies even reported a protective effect of opium on CAD [8] and cardiovascular outcomes [22,23]. On the other hand, most existing literature emphasizes the detrimental effects of opium on accelerating atherosclerosis and worse CAD [24]. Nevertheless, the magnitude of the impact of opium use on the prognosis and cardiovascular long-term outcomes of patients with established ischemic heart disease was yet to be determined. Hence, our study followed a large number of patients with established CAD for approximately 10 years and provided evidence favoring a 27%–57% increase in the risk of major cardiovascular events among opium users compared to non-users. A similar finding was observed by Masoudkabir et al. [25], who studied a large number of patients who underwent coronary artery bypass graft surgery and showed that opium users had a 25% higher risk of major adverse cardiovascular events compared to non-users during a long-term follow-up period. Hence, we believe these long-term effects are most likely driven by a higher risk of death and non-fatal myocardial infarction after the index procedure among opium users compared to non-users. There even seems to be a dose-response increase in the risk of myocardial infarction with higher amounts of opium consumption based on the available evidence [26].

Despite the observed relationships between opium use and poor health outcomes, the exact mechanisms by which opium consumption results in such an increased risk of adverse cardiovascular events are not clearly defined. It is suggested that opium use generates an inflammatory state by inducing pro-inflammatory mediator release, reducing anti-inflammatory cytokines, and inducing systemic oxidative stress, thereby leading to higher levels of inflammatory biomarkers, pro-coagulant factors, and higher degrees of insulin resistance [27,28,29,30]. In addition, the pharmacodynamics and pharmacokinetic properties of antiplatelet agents may be altered with the use of opium or its derivatives [31,32]. Nonetheless, more studies are still needed to elucidate the mechanisms behind the adverse cardiovascular effects of opium.

Opium users in our study seem to be younger and less likely to have traditional cardiovascular risk factors, except for a higher prevalence of cigarette smoking. This is consistent with the risk factor profile observed in previous studies that investigated opium users with more extensive CAD [25,33]. Although there are some traditional beliefs that opium use has cardiovascular and health benefits, evidence-based data suggest that opium consumption does not improve conventional cardiovascular risk factors [34]. Opium users tended to present with myocardial infarction at a younger age compared to non-users, rendering opium consumption a major cardiovascular risk factor in those with premature CAD, especially in younger men [33,35]. Furthermore, opium use was significantly associated with a higher risk of premature all-cause mortality in a long-term follow-up study [36].

Overall, special attention should be paid to the assessment of opium consumption status in patients with established CAD. This is of utmost importance among candidates for revascularization, particularly younger men with premature CAD. These patients should be closely followed to ensure compliance with medications, should undergo risk factor modification, and attempting to wean patients off opioids. These preventive strategies for discontinuation of opium use should be widely implemented to mitigate the long-term risk of adverse cardiovascular events after revascularization.

### Limitations

This study is mainly limited by its single-center retrospective nature. Although we included a large number of consecutive patients, further studies are warranted to evaluate the generalizability of our findings. Similar to many previous studies investigating habitual risk factors, opioid use was self-reported, and the possibility of recall bias and underreporting of opioid use cannot be ruled out. Since the present study was not explicitly designed to capture detailed information on opium consumption habits, we could not integrate the duration and dosage of opium use into our analyses. Furthermore, we did not have comprehensive data regarding the use of opioid-derived medication prescriptions to include in our definition of opium use. Although we used a robust statistical model to adjust for multiple potential confounders and account for between-group imbalances, some degree of bias still cannot be excluded entirely due to the possibility of residual confounding. Lastly, the observed relationships with opium consumption should be interpreted as mere associations rather than drawing causal inferences.

## Conclusion

Opium consumption in patients undergoing elective PCI is associated with a more than 50% increase in long-term risk of overall mortality and MACCE. These harmful effects of opium use are most likely due to an elevated risk of death and non-fatal myocardial infarctions. Therefore, a history of opioid substance abuse should be thoroughly sought and targeted in patients with established CA to offer timely preventive measures.

## Data availability statement

The data underlying this article will be shared on reasonable request to the corresponding author.

## Additional File

The additional file for this article can be found as follows:

10.5334/gh.1315.s1 Supplementary Figure S1. Propensity score coverage before and after inverse propensity score weighting.
