# Supplementary material for: Long-Term Effects of Opium Consumption Following Percutaneous Coronary Intervention: A 10-year Follow-Up Study
Source: Glob Heart. 2024 Apr 24;19(1):38. doi: 10.5334/gh.1315 (PMC11049677; doi:10.5334/gh.1315)

Supplementary Figure S1. Propensity score coverage before and after inverse propensity score weighting

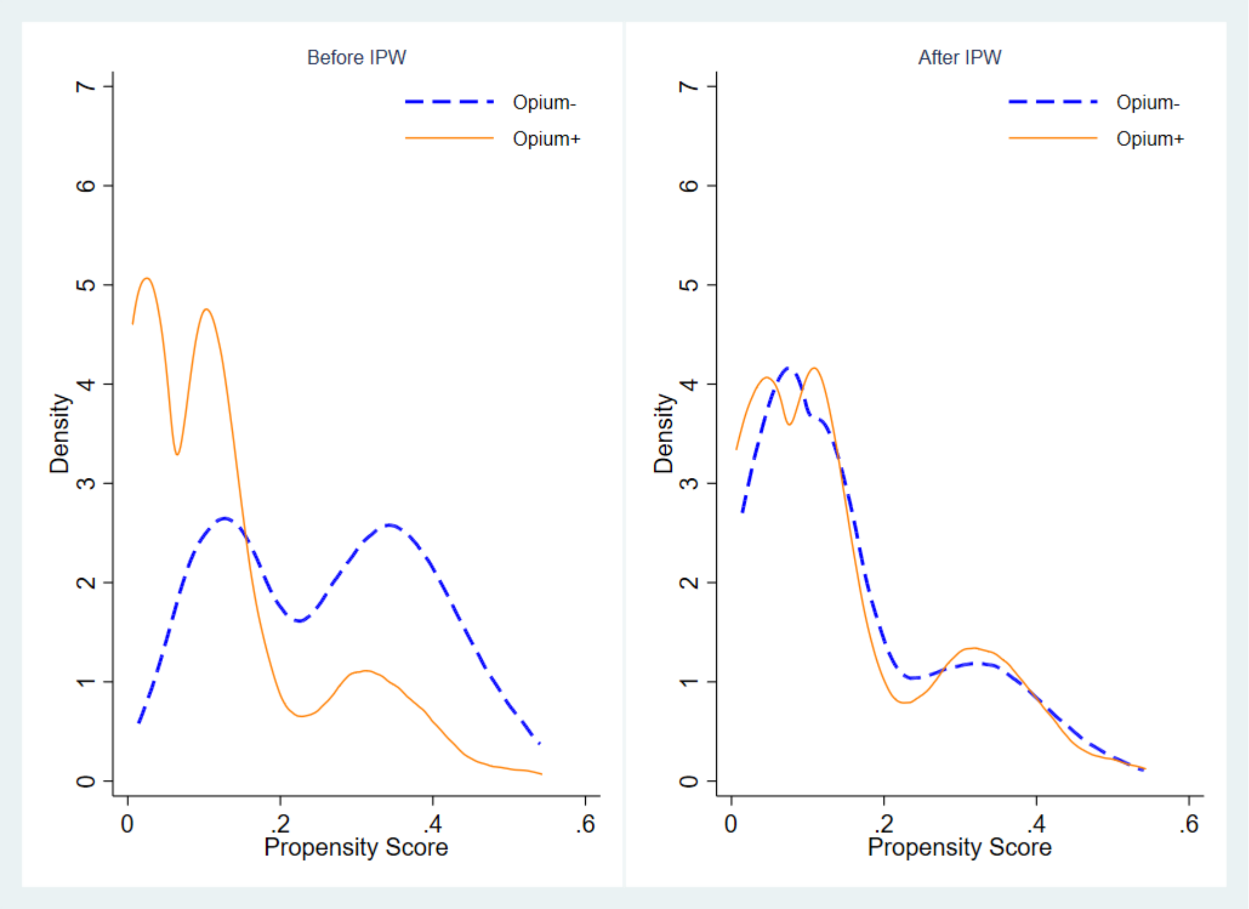

Supplement: Supplementary Figure S1. — Propensity score coverage before and after inverse propensity score weighting. [file gh-19-1-1315-s1.pdf]
